# Assessing Genomic Diversity and Signatures of Selection in Chinese Red Steppe Cattle Using High-Density SNP Array

**DOI:** 10.3390/ani13101717

**Published:** 2023-05-22

**Authors:** Mingyue Hu, Hao Jiang, Weining Lai, Lulu Shi, Wenfeng Yi, Hao Sun, Chengzhen Chen, Bao Yuan, Shouqing Yan, Jiabao Zhang

**Affiliations:** College of Animal Science, Jilin University, Changchun 130062, China

**Keywords:** Chinese Red Steppe Cattle, genetic diversity, population structure, selection signatures, SNP chip

## Abstract

**Simple Summary:**

The Chinese Red Steppe Cattle (CRS) is a well-known dual-purpose (meat and milk) cattle breed. Here, the genetic variation and population structure of CRS were studied using 100 K SNP genotyping data. The results show that the genetic structure of CRS is different from other populations, the level of genetic diversity is high, and the level of inbreeding is low. In conclusion, our research provides the genetic basis for the prominent characteristics of CRS, which can be used to improve the breeding program of CRS in the future.

**Abstract:**

Chinese Red Steppe Cattle (CRS), a composite cattle breed, is well known for its milk production, high slaughter rate, carcass traits, and meat quality. Nowadays, it is widely bred in Jilin and Hebei Province and the Inner Mongolia Autonomous region. However, the population structure and the genetic basis of prominent characteristics of CRS are still unknown. In this study, we systematically describe their population structure, genetic diversity, and selection signature based on genotyping data from 61 CRS individuals with GGP Bovine 100 K chip. The results showed that CRS cattle had low inbreeding levels and had formed a unique genetic structure feature. Using two complementary methods (including comprehensive haplotype score and complex likelihood ratio), we identified 1291 and 1285 potentially selected genes, respectively. There were 141 genes annotated in common 106 overlapping genomic regions covered 5.62 Mb, including *PLAG1*, *PRKG2*, *DGAT1*, *PARP10*, *TONSL*, *ADCK5*, and *BMP3*, most of which were enriched in pathways related to muscle growth and differentiation, milk production, and lipid metabolism. This study will contribute to understanding the genetic mechanism behind artificial selection and give an extensive reference for subsequent breeding.

## 1. Introduction

Domestication and selective breeding is the process by which wild individuals are bred in captivity and modified through artificial selection to be phenotypically and genetically distinct from the original ancestors [1,2]. Modern domesticated cattle can be categorized into two subspecies: *Bos taurus indicus* (indicine or zebu) and *Bos taurus taurus* (taurine), according to their different morphological characteristics and living habits [3]. With the expansion of agricultural society and human activities, extensive blending has occurred between different taurine and indicine populations, so there are more than over 800 live cattle breeds in the world [4]. Hybridization is a common strategy for the formation of modern livestock breeds and improving the undesirable performance of some cattle breeds, which leads to the superiority of a heterozygote over their purebred parental breeds in one or more traits [5]. In China, breeding specialized breeds through crossbreeding with commercial breeds has become an important way to improve the production efficiency of Chinese indigenous breeds. It is necessary to evaluate the genetic diversity and observe the development status of these composite breeds.

With the availability of single nucleotide polymorphism (SNP) genotyping data, genome-wide SNP chips have been widely used to study genetic diversity, population structure, inbreeding level, effective population size, genome-wide association study, and selection feature detection in different breeds or populations worldwide [6,7]. At present, depending on the SNP chips, several studies on the evaluations of genetic relatedness have been carried out in farm animals such as cattle, pigs, and dogs, and many remarkable scientific achievements have been achieved [8,9,10]. In cattle, many studies focused on the economic traits under the positive selection of commercial breeds and the adaptability of indigenous breeds [11]. The Bovine HapMap Consortium interrogated 37,470 SNPs in 497 cattle from 19 geographically and biologically diverse breeds and found that domestication and artificial selection appear to have left detectable signatures of selection within the cattle genome [12]. Chen. et al. used a 77 K chip to analyze the origin of Qinchuan cattle and found that it belonged to the mixed origin of taurine and indicine and was accompanied by a small amount of Javan cattle ancestry [13].

After long-term selection, breeds living in different environmental conditions have formed a unique adaptive evolutionary system [14]. Differences in genomic structural features caused by artificial or natural selections are known as selection signals related to the direction of reproduction and the mechanism of domestication. Therefore, the detection of selection signals is vital to uncover the genes related to economic traits and explore the adaptation mechanism of domestication. In recent years, several statistical methods have been used to detect recent selection footprints in composite populations, including comprehensive haplotype score (iHS), complex likelihood ratio (CLR), cross-population extended haplotype homozygosity (XP-EHH), and interpopulation relatively comprehensive haplotype homozygosity (Rsb) [15,16]. For example, based on the Illumina BovineLD v2 BeadChip data, van der Nest. et al. identified 10 candidate regions and genes that are potentially under strong positive selection using the iHS and Rsb methods in an admixed South African Simbra crossbred population [5]. Ma. et al. identified potentially selected genes at the genome-wide level in Huaxi cattle, which related to ion binding and muscle growth and differentiation [15].

Chinese Red Steppe Cattle (CRS), the first composite breed for both milk and meat purposes since the founding of China, was developed by crossbreeding Shorthorn with Chinese native Mongolian cattle [17]. The breeding work of CRS started in 1958, and the new variety was identified by the National Breeding Committee and officially named in 1985 [18,19,20]. It is characterized by its excellent production performances, including high milk production, growth and carcass traits, strong stress resistance, and cold climate adaptation, and is widely farmed, especially in northeast China [21,22]. Evaluating population genetic characteristics and selection signatures can provide important insight into the genetic relationship and molecular background of special phenotypes among breeds. In this study, using Illumina GGP Bovine 100 K genotyping data, the population structure, genetic diversity of CRS, and relationship with other commercial and native breeds were analyzed. Additionally, selective sweep analysis was carried out to detect candidate regions and potential genes underlying the carcass and milk characteristics of CRS. Our study will help to promote the conservation and sustainability of CRS and advance understanding of the mechanisms underlying the formation of specific traits in CRS.

## 2. Materials and Methods

### 2.1. Sample Selection

Blood samples were collected from 61 CRS individuals randomly selected from the Sanjiazi Cattle Breeding Farm of Tongyu Country, Jilin Province. Cattle blood DNA was extracted with a kit from Beijing Tiangen Biotechnology. After extraction, the integrity of genomic DNA was detected with 1.0% agarose gel. The quality and quantity of the DNA were estimated using a Nanodrop 2000 nucleic acid protein analyzer (Thermo Scientific, Wilmington, NC, USA). The obtained DNA bands are neat and clear without trailing, and the purity of DNA samples (OD260/OD280 = 1.8–2.0) means less degradation and less protein residue. In addition, the re-sequencing data of 245 individuals from 15 breeds were downloaded from the NCBI database on 13 October 2022. (https://www.ncbi.nlm.nih.gov/sra/); its parental ancestors include Shorthorn cattle (SHO, n = 20), Mongolian cattle (MON, n = 27), and Angus cattle (ANG, n = 22), Hereford cattle (HER, n = 20), Holstein cattle (HOL, n = 20), Charolais cattle (CHA, n = 23), Limousin cattle (LIM, n = 20), Simmental cattle (SIM, n = 27), Tibetan cattle (TIB, n = 11), Kazakh cattle (KAZ, n = 11), as well as five local Chinese populations, Yanbian cattle (YAN, n = 11), Luxi cattle (LUX, n = 7), Lingnan cattle (LIN, n = 8), Wenling cattle (WEN, n = 11), Zhoushan cattle (ZHO, n = 7). Sample details are presented in Tables S1 and S2.

### 2.2. SNP Genotyping and Quality Control

Samples of 61 CRS were genotyped using the GGP Bovine 100 K Chip. All re-sequencing data of 245 individuals were aligned to the cattle reference genome (ARS-UCD1.2) using BWA with command ‘bwa mem’, and SNP calling was performed following the GATK pipeline v4.4.1.0 [23,24]. Then, we obtained the high-quality raw SNPs by using the module VariantFiltration with the parameters ‘QD < 2.0, FS > 60.0, MQ < 40.0, MQRankSum < −12.5, ReadPosRankSum < −8.0 and SOR > 3.0′ of GATK. Finally, the genotyping data of the CRS were merged with the re-sequencing data using the PLINK v1.9 ‘--merge’ command [25]. Only SNPs located on autosomal were used for subsequent analysis, and SNP was assessed using PLINK with option ‘--chr 1—29′. To increase the data processing accuracy, PLINK v1.9 software was adopted for quality control. SNPs were obtained after the exclusion of those with either call rate ≤95% and minor allele frequency (MAF) ≤3% using the command ‘--geno 0.05′ and ‘--maf 0.03′. After that, PLINK v1.9 ‘--mind 0.1′ command was used to remove samples with genotype loss rate greater than 10%, and the remaining SNPs were used for further analysis.

### 2.3. Genetic Diversity 

In order to assess genetic diversity, the average observed heterozygosity (*H*_O_) and expected heterozygosity (*H*_E_) were estimated using PLINK v1.9 with option ‘--hardy’ [26]. The nucleotide diversity (pi) was estimated using VCFtools with the parameters ‘--window-pi 10,000 --window-pi-step 5000′. In addition, to assess the inbreeding degree, the homozygosity inbreeding coefficient (*F*_HOM_) was calculated using PLINK v1.9 command ‘--het’ [25], and the total length of the ROH fragment was calculated using the “--homozyg” command setting [27,28]. After that, the runs of the homozygosity-based inbreeding coefficient based on ROH (*F*_ROH_) were calculated by the total length of the ROH fragment divided by the length of the autosomal genome.

### 2.4. Population Structure Analysis

To ascertain the genetic relationship between CRS and other breeds, PLINK v1.9 was used to remove those sites with high LD with the parameter ‘-indep-pairwise 50 25 0.2′ [25], and the remaining 39,814 SNPs were used for population structure analysis. Principal component analysis (PCA) was performed using GCTA v1.92.3beta3 to discern genetic relationships among breeds [29]. The graphical representation of PCA was depicted using the plot function in R3.6.1. In addition, admixture analyses using ADMIXTURE 1.30 software with the parameters ‘admixture --cv’ were implemented to validate the cluster patterns among our dataset [30]. The corresponding cross-validation error value for clustering (*K* = 2 to 9) was also calculated in ADMIXTURE, and the graphical representation of the ADMIXTURE results was performed using R script, as suggested by the ADMIXTURE procedure. 

### 2.5. Identification of Selection Signature

In order to detect selection signatures under the recent or ongoing positive selection in the CRS genome, the selection sweeps were identified based on two complementary methods: the Integrated Haplotype score (iHS) and composite likelihood ratio (CLR) approach, respectively. In this study, BEAGLE v5.2 with the parameters ‘beagle gt = test.vcf out = test.beagle.vcf ne = 306′ was used to impute missing alleles and infer the haplotype phase for all individuals [31]. The mean iHS value was calculated by 100-kb non-overlapping window across the autosomes and the norm module of selscan was applied to normalize the iHS score [32]. The density of signal in each region was evaluated according to the proportion of SNPs with |iHS| > 2, which essentially reflected unusually long haplotypes across the genome [33]. Finally, the regions under selection as those with the top 5% highest average |iHS| score were regarded as candidate regions of positive selection. The CLR test was calculated for sites in non-overlapping 100-kb windows using the software SweeD v3.2.1 [34]. To define candidate regions, the maximum CLR value was used as the test statistic according to previous studies [35], and the top 5% CLR values were selected as candidate regions in this study. In order to reduce false-positive regions, the overlap of genomic regions identified by the two methods with outlier signals (top 5%) was considered as candidate signatures of selection [11].

### 2.6. Enrichment Analyses of Candidate Genes under Selection

Based on the bovine ARS-UCD1.2 reference genome annotation, we retrieved genes with candidate significant of SNP loci under selection. Furthermore, gene function was determined using the National Center for Biotechnology Information database accessed on 14 November 2022 (https://www.ncbi.nlm.nih.gov/). In this study, enrichment analyses for genes within overlapping significant candidate regions detected using the iHS method (top 5%) and the CLR method (top 5%) were performed. To gain a better understanding of the gene functions and signaling pathways of the identified candidate genes, Gene Ontology (GO) and Kyoto Encyclopedia of Gene and Genomes (KEGG) pathway enrichment analysis were performed using KOBAS [36]. Thus, the significantly enriched pathways were considered to be significantly enriched only when the corrected *p*-value was less than 0.05 [37]. 

### 2.7. Aligning Core Regions to QTL Database

Moreover, the cattle quantitative trait locus (QTL) database was used to identify the overlapping regions associated with the most plausible trait-associated selective signatures. We used the bovine database incorporated in the Animal QTL database to determine the potential overlap of these regions [38]. The number and function of candidate regions were determined after annotation.

## 3. Results

### 3.1. SNP Genotyping and Genetic Diversity

In total, 245 individuals from 15 breeds were generated and aligned to the *B.taurus* reference genome ARS-UCD1.2, and 61 individuals from CRS were successfully genotyped with the GGP Bovine 100 K Chip. Furthermore, a total of 82,101 common autosomal bi-allelic SNPs were detected in the merged data across 306 individuals; out of 80,871 SNPs remaining after the removal SNPs with <95% call rate, MAF < 0.03 with an average distance of 32.3 kb distributed over 29 chromosomes were obtained for subsequent analysis. The length of each chromosome, number, percentage of SNPs, and the average interval between SNPs for each chromosome are shown in Table S3. The results showed that the highest number of SNPs (4994 SNPs) was found on chromosome 1, and the lowest (1523 SNPs) was found on chromosome 25. The distribution of SNPs on each chromosome within 1 Mb window size is shown in Figure S1, and the result showed that SNPs are evenly distributed across the chromosome. The average observed heterozygosity (*H*o), average expected heterozygosity (*H*e), the homozygosity inbreeding coefficient (*F*_HOM_), and inbreeding coefficient based on ROH (*F*_ROH_) have been calculated to assess polymorphism of 16 cattle populations. The results indicated that the pi value ranged from 0.000034 to 0.000047 (Table S4). Among them, CRS (0.000043) was higher than that in most indicine breeds but lower than that in most taurine breeds. Among all groups in this study, the *H*_O_ of CRS (0.387) was higher than most commercial breeds, including Simmental (0.386), Angus (0.377), and Holstein (0.372), and the *H*_E_ of CRS (0.375) was between Hereford and Angus. Overall, these results indicated high genetic diversity in CRS. As shown in S3, the results showed that the *F*_HOM_ (−0.0312) in CRS was very close to zero. In addition, the *F*_ROH_ value of CRS was 0.088, which was lower than the three commercial cattle population of Hereford cattle (0.133), Angus cattle (0.111), and Holstein cattle (0.096), and approximately that of local Chinese cattle of Lingnan cattle (0.073), and Zhoushan cattle (0.079). The results indicating the existing breeding programs effectively avoid inbreeding.

### 3.2. Population Structure and Admixture Analysis

**Figure 1:**
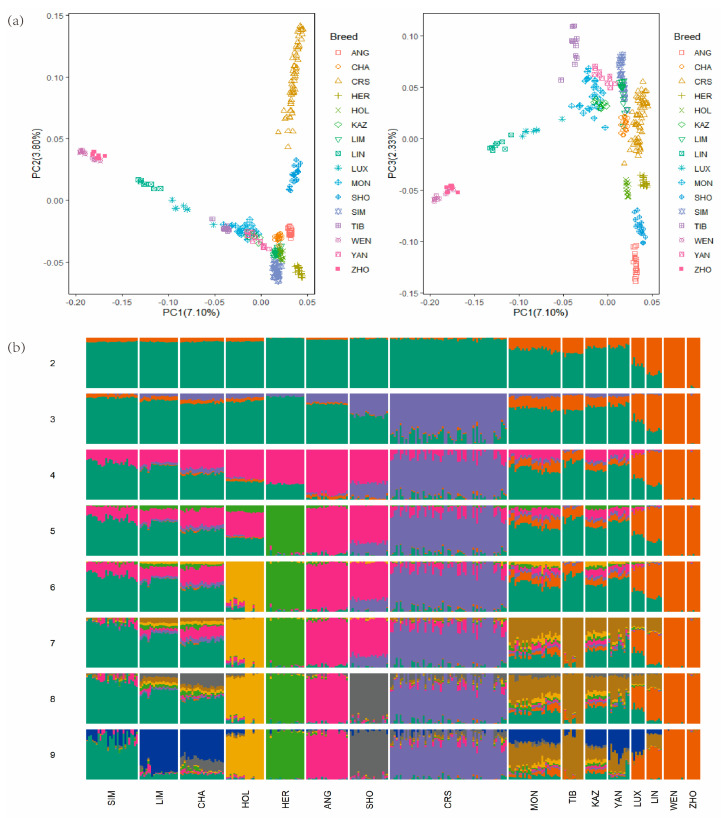
Population structure and relationships of CRS compared with other breeds. (**a**) The PCA result of 306 individuals from 16 cattle populations. (**b**) Model-based clustering among different cattle using ADMIXTURE (*K* = 2–9). Abbreviations: CRS, Chinese Red Steppe Cattle; ANG, Angus; HER, Hereford; SIM, Simmental; LIM, Limousin; CHA, Charolais; HOL, Holstein; SHO, Shorthorn; MON, Mongolian; TIB, Tibetan; KAZ, Kazakh; YAN, Yanbian; LUX, Luxi; LIN, Lingnan; WEN, Wenling; ZHO, Zhoushan. Individuals were shown as a thin vertical line colored in proportion to their estimated ancestry.

To investigate the cluster patterns among the CRS cattle and other cattle breeds, we conducted PCA and ADMIXTURE using genomic SNPs. The genetic relationships among the 16 cattle breeds revealed using PCA are shown in Figure 1. The first and second PCs explained 7.10% and 3.80% of the variation in the entire genomic data, respectively (Figure 1a). In particular, the first PC obviously separated CRS and SHO from other breeds, which had the greatest explanatory power. Next, to determine the admixture degree in the 16 cattle populations, ADMIXTURE software was used to infer the proportions of individuals in CRS cattle. The hypothetical ancestral groups ranged from *K* = 2 to 9, with the lowest cross-validation error value being 9 (Figure 1b). When *K* = 2, these different cattle breeds can be genetically divided into two groups: *Bos taurus* and *Bos indicus*. When *K* = 3, CRS cattle displayed different admixture component proportions among SHO and MON populations; it is more similar to SHO, which indicates that the genetic influence of SHO cattle was greater than that of MON cattle, a result consistent with the principal component analysis result. When *K* = 8, CRS displayed different admixture component proportions among all populations, indicating that it formed its own unique genetic features obviously distinguished from all other populations. 

### 3.3. Identification of Selection Signatures

**Figure 2:**
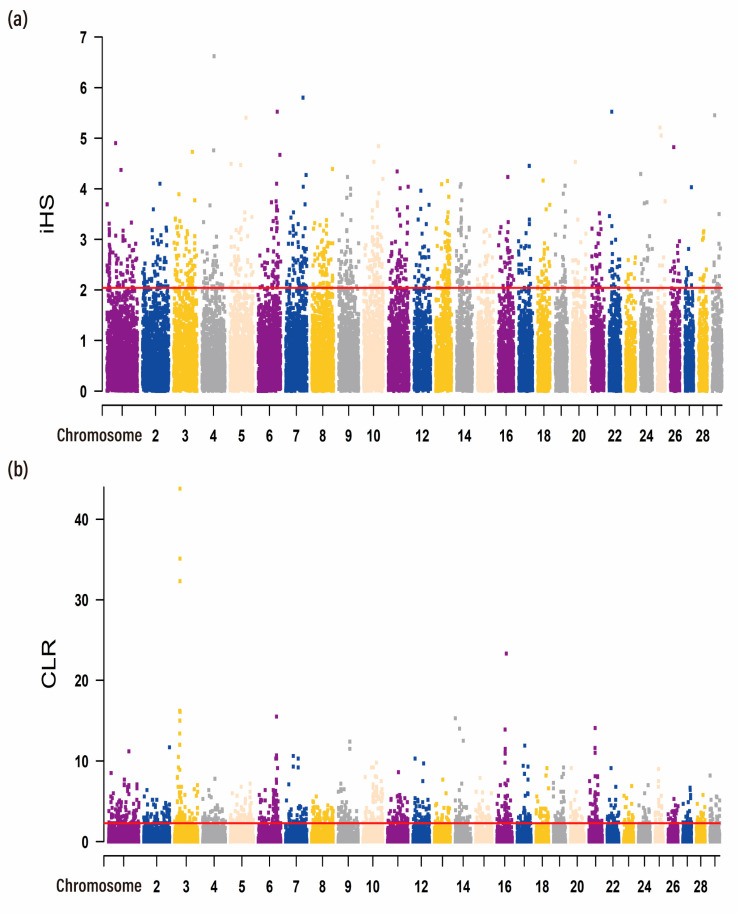
Genome-wide distribution of selection signatures detected by iHS and CLR in CRS. Red line displays the threshold levels of 5%. (**a**) Manhattan plot of iHS scores in CRS. (**b**) Manhattan plot of CLR scores in CRS. Alternating colors distinguish markers on neighboring chromosomes.

In this study, we applied the iHS and CLR methods to detect the genomic regions under the recent selection of the CRS. Two methods showed outlier signals (top 5%) in overlapping regions and were, therefore, considered candidate selective regions. The genome-wide distribution of |iHS| values and CLR values for 100 k non-overlapping windows on autosomes is depicted in Figure 2. Results of the |iHS| value indicated that the distribution of selective signatures was not uniform across the genome; the average |iHS| value was 0.76, and the maximum |iHS| value was 6.62. In total, there were 1122 candidate regions under the threshold of the top 5% that were identified, and we obtained 1291 genes under selection in the iHS test (Figure 2a; Table S5). In addition, We identified 1235 regions meeting the top 5% CLR values and were selected as candidate regions with a total of 1285 genes (Figure 2b; Table S6). Through gene retrieval, 141 potentially selected candidate genes were detected in 106 overlapping genomic regions covered 5.62 Mb by both methods, indicating strongly selected in the CRS population (Table S7). The commonly detected regions showed several candidate genes already reported as selection signals or related to excellent economic traits, such as milk yield and lactation performance (*DGAT1*, *OPLAH,* and *GRINA*) [39,40,41], lipid metabolism (*PARP10* and *AMFR*) [42,43], and growth and carcass traits (*PRKG2*, *ZFP90,* and *BMP3*) [44,45]. Among them, most of these genes were located on chromosomes 10 and 14, respectively (Table 1 and Table S7). Notably, *PLAG1* has been fully proven to be associated with stature and body size, which may affect the beef production of CRS [46,47,48]. *PRKG2* plays a role in growth and carcass [49,50]. *DGAT1* showed some positive role in the enhancement of meat and carcass fatness quality in beef cattle and obtained considerable attention, especially in animal milk production [51].

### 3.4. Gene Annotation and Enrichment Analysis

The functional enrichment analysis using KEGG pathways and Gene Ontology (GO) for overlapped genes were further analyzed. The significantly enriched GO terms and KEGG pathways are shown in Table S8. Among them, a total of 290 significantly enriched GO terms with corrected *p*-value < 0.05 were observed, such as regulation of cell cycle (GO:0051726, *p* = 0.00681), identical protein binding (GO:0042802, *p* = 0.000112), protein-containing complex binding (GO:0044877, *p* = 0.000349), and positive regulation of protein catabolic process (GO:0045732, *p* = 0.0246). In addition, metabolism-related and immune-related biological functions were significant. Furthermore, eighteen significant enriched pathways were obtained, including the mRNA surveillance pathway (bta03015, *p* = 0.00113), protein processing in the endoplasmic reticulum (bta04141, *p* = 0.00769), cGMP-PKG signaling pathway (bta04022, *p* = 0.0447), and cAMP signaling pathway (bta04024, *p* = 0.0222), which are related to metabolism and protein synthesis. 

Moreover, the cattle quantitative trait locus (QTL) database was used to identify the overlapping regions associated with the most plausible trait-associated selective signatures. In total, 436 QTLs were located within or overlapping with these 106 candidate regions (Table S9). Most of these regions have been found to be related to economically important traits in cattle, including milk traits, meat and carcass traits, and reproduction traits. Notably, 106 (24.3%) QTLs belonging to 77 candidate regions were associated with milk traits, and 99 (22.7%) QTLs belonging to 95 candidate regions were associated with meat and carcass traits, suggesting the strong selection for milk and meat traits during the breeding of CRS.

## 4. Discussion

CRS is a crossbred cattle breed in China, and it has been intensively bred for beef over the past 30 years, with characteristics of high slaughter rate, excellent meat quality, greater disease resistance, and good reproductive performance [17]. The characterization of genetic diversity, population structure, and genetic relationships at the genome-wide level is essential for evaluating bovine genetic resources and uncovering genetic divergence, as well as detecting the selection signatures [52,53]. The data generated from this study will help inform and design appropriate management and breeding strategies to maximize the productivity of CRS. The dataset contains a total of 82,101 SNPs in 306 individuals from 16 breeds and 80,871 SNPs with an average distance of 32.3 kb distributed over 29 autosomal chromosomes after quality control. Among all groups in this study, the observed heterozygosity, expected heterozygosity, and pi of CRS were 0.387, 0.375, 0.000043, respectively, which were at the relatively high level of genomic diversity among the 16 cattle populations in this study. One reason may be that CRS was crossed with local breeds, which is consistent with previous research [54]. On the other hand, the bovine SNP chips were optimized for use in taurine, which has a little bias and is not suitable for studying the genetic diversity and origin of domestic cattle in other regions [55]. The GGP Bovine 100 K Chip can provide the amount of neutral information to adapt to most breeds and is widely used for genetic diversity and selection signal analysis of local or cultivated varieties [56,57]. Although crossbreeding is still an important method to increase the genetic variation of modern cattle breeds, high-intensity artificial selection may promote the improvement of population inbreeding levels and, thus, reduce the genetic diversity of breeds, which should be noted in the process of breeding [58]. The results indicated that the *F*_HOM_ and *F*_ROH_ of CRS exceeded the expected value, which was lower than a lot of commercial cattle populations. In this study, the genetic diversity of CRS holds significant potential for improvements in production. Therefore, based on the existing breeding, breeding cattle with a large genetic distance should be selected to increase the population size and maintain the genetic diversity of the CRS population. In the future, we could obtain more genomic information using whole-genome data to more precisely elucidate the genetic diversity and represent a realistic estimate of total genomic inbreeding in CRS [59,60]. 

PCA and ADMIXTURE were used to investigate the genetic relationship and population-level admixture among CRS and other breeds. The results indicated that the genomic background of the CRS breed initially represents the fusion of ancestral varieties. In addition, CRS individuals were obviously separated from other cattle breeds, and the CRS breed composition stabilized over time after initial crossbreeding and subsequent artificial selection. 

The domestication of various livestock species is one of the greatest achievements of humankind [61]. In the process of domestication, domesticated animals have been subjected to multiple natural selections such as environment, food, and pathogens, as well as strong artificial selection in terms of economic traits. With the maturity and rapid development of second-generation sequencing technology, more and more sequencing data have been accumulated, which can detect the selection footprint left on the genome in a large range [62,63]. By analyzing the selection signatures in the genome, we can understand the evolutionary history of the population and better guide the study of biological evolution and genetic breeding [64,65]. With the improvement in analytical methods and strategies, as well as more consideration of population factors, many approaches have been proposed to identify selection signals, such as iHS, CLR, XP-EHH, and *F*st. In this study, the iHS and CLR were used to identify candidate genomic regions under positive selection with important traits in CRS [66,67]. We identified 1291 and 1285 potentially selected genes in CRS, respectively, of which 5.62 Mb selection area overlapped in the two selective signature detection methods. Complex quantitative traits, such as milk traits, are controlled by numerous genes with small effects [68,69]. Previous studies suggested that *DGAT1* was documented to have a significant influence on milk production in cattle in Germany and is associated with milk production traits through GWAS [70,71]. Of these candidate genes, a series of genes (*DGAT1*, *TONSL*, *CPSF1*, *ADCK5*, *GRINA*, and *PARP10*) in the CRS that has the strongest signal for milk production traits also reported various levels of interactions in which *CPSF1* as the hub gene since it interacted with all the other genes [51]. Additionally, *TONSL*, *CPSF1*, *ADCK5*, and *DGAT1* genes are neighbors and there are coexpressions among each other [72]. Moreover, several of the candidate genes found for body size were previously reported in humans, cattle, goats, and horses [73,74,75,76,77]. Variation in the genome region coding for *PLAG1* has well-documented associations with skeletal growth and age at puberty in cattle [78], and it has been reported in humans that *PRKG2* gene deletion is associated with growth restriction [79,80]. In this study, many results highlighted genes that improve milk, meat, and lipid metabolism and their quality characteristics. The heritability of these traits increases due to the frequency of dominant mutations of genes affecting traits in the population, which leads to a positive selection of gene regions [16]. Moreover, QTL associated with milk yield and meat production traits were significantly enriched in selection signatures. Overall, our results demonstrate the genetic basis of economically important traits, show evidence of sustained selection, and provide a reference for subsequent breeding strategies.

## 5. Conclusions 

The results presented in this study indicated that CRS cattle have high genetic diversity, a low level of inbreeding, and have formed unique genetic features. Meanwhile, putative selection regions containing candidate genes associated with the traits of milk production, growth, and carcass traits were also identified and annotated. These findings will be very useful for conservation, management, and selection approaches in CRS cattle in the future.

## Figures and Tables

**Table 1:** Potential selected genes associated with important economic traits in CRS.

Chr	Position (bp) ^a^	Candidate Genes	Traits
6	95,951,816–96,039,423	*PRKG2*	Meat and carcass
14	22,865,966–22,878,026 22,878,513–22,965,966	*XKR4*	Feed intake and growth
14	23,280,460–23,365,970	*PLAG1*	Body height trait
14	771,431–865,745	*GRINA*	Milk yield and lactation
14	670,944–770,944 771,431–865,745	*OPLAH*	Milk yield and lactation
14	570,458–665,743	*DGAT1*	Milk and Meat Production
14	469,971–569,971 570,458–665,743	*ADCK5*	Milk production
14	469,971–565,742	*TONSL*	Milk production
14	469,971–565,742	*CPSF1*	Milk production
14	771,431–865,745	*PARP10*	Lipid metabolism
14	469,971–565,742	*VPS28*	Milk fat synthesis
14	469,971–565,742	*CYHR1*	Milk production and lactation
14	670,944–765,744	*MAF1*	305-day milk
18	24,199,314–24,261,243	*AMFR*	Lipid metabolism
18	35,861,360–35,909,418	*ZFP90*	Growth traits
1	148,529,301–148,616,272	*DOP1B*	Fertility trait
14	771,431–865,745	*SPATC1*	Fertility trait
6	95,750,518–95,850,518	*BMP3*	Skeletal development
14	670,944–765,744	*EXOSC4*	Embryonic lethality

^a^ This column presents the position of candidate genes that are within or overlap with the potential regions of selection.

## Data Availability

The genotyping data supporting the findings of the present study are available in FigShare (https://doi.org/10.6084/m9.figshare.21586182.v1), and the file(s) become available on 19 November 2024.

## Supplementary Materials

The following supporting information can be downloaded at: https://www.mdpi.com/article/10.3390/ani13101717/s1. Figure S1: Density distribution map of SNPs on chromosome within 1 Mb window size; Table S1: Populations and number of samples used in the study; Table S2: Whole-genome sequencing (WGS) data used in this study; Table S3: Number of SNPs in each autosome for merge data in 306 individuals from 16 breeds; Table S4: Genetic diversity of the 16 cattle breeds used in this study; Table S5: A summary of genes from iHS in CRS; Table S6: A summary of genes from CLR in CRS; Table S7: 106 overlapping genomic regions; Table S8: GO and KEGG enrichment analysis of CRS candidate genes by iHS and CLR methods; Table S9: QTLs overlapped with candidate selected regions.
