# Supplementary figures and images for: Assessing Genomic Diversity and Signatures of Selection in Chinese Red Steppe Cattle Using High-Density SNP Array
Source: Animals (Basel). 2023 May 22;13(10):1717. doi: 10.3390/ani13101717 (PMC10215729; doi:10.3390/ani13101717)

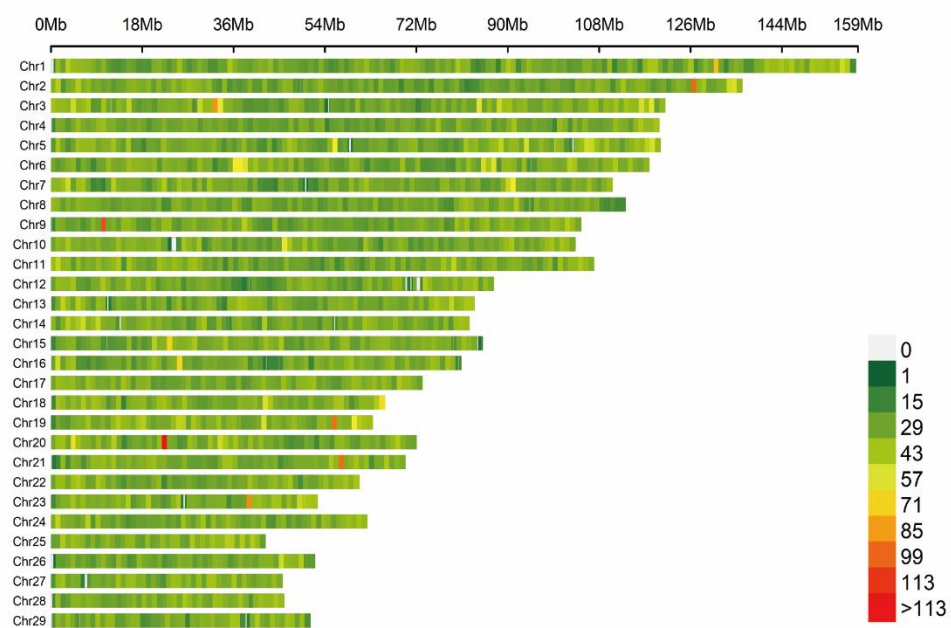

Fig.S1 Density distribution map of SNPs on chromosome within 1 Mb window size

Supplement: Supplementary file 1 [file animals-13-01717-s001.zip › Fig.S1 Density distribution map of SNPs on chromosome within 1 Mb window size.pdf]
